# Evaluation of cognitive impairments in multiple sclerosis: a comparative analysis of the Rey complex figure test and the symbol digit modalities test

**DOI:** 10.1055/s-0046-1817028

**Published:** 2026-03-11

**Authors:** Mesrure Köseoğlu, Aslı Yaman Kula, Mehmet Demir, Ebru Temiz, Betül Artun, Zeynep Aksu, Gürkan Yaman, Nazlı Durmaz Çelik, Serkan Özben

**Affiliations:** 1 Kanuni Sultan Suleyman Training and Research Hospital, Department of Neurology, Istanbul, Turkey.; 2 Eskişehir Osmangazi University Medical Faculty Hospital, Department of Neurology, Eskişehir, Turkey.; 3 Antalya Training and Research Hospital, Department of Neurology, Antalya, Turkey.

**Keywords:** Multiple Sclerosis, Cognitive Dysfunction, Neuropsychological Tests

## Abstract

**Background:**

Cognitive impairment is common in multiple sclerosis (MS), and visual memory impairments are increasingly considered clinically important, yet still underappreciated.

**Objective:**

To compare cognitive performance assessed by the Rey Complex Figure Test (RCFT) and Symbol Digit Modalities Test (SDMT) in MS patients and healthy controls, and to examine correlations between these measures and physical disability indices.

**Methods:**

A total of 177 participants were assessed using RCFT subtests and SDMT, of whom 95 had relapsing-remitting MS (RRMS) and 82 were healthy controls. Group comparisons and correlation analyses were performed.

**Results:**

The MS patients scored significantly lower on all RCFT subtests and SDMT. Moderate correlations were found between SDMT and RCFT 0 to 2, but not RCFT 3.

**Conclusion:**

Both RCFT and SDMT may serve as potentially complementary measures of cognitive domains relevant to MS. Incorporating both recall and recognition-based measures, along with processing speed assessments, can provide a broader evaluation of cognitive strengths and weaknesses in MS, helping clinicians better characterize patients' profiles.

## INTRODUCTION

Multiple sclerosis (MS) is a demyelinating and inflammatory disease of the central nervous system. 1 2 3 4 Cognitive impairments are known to emerge in its early stages and progressively worsen over time in these patients. Cognitive dysfunction significantly impacts the quality of life and functionality of patients, thereby increasing the overall burden of the disease. This underscores the importance of early and precise evaluation of cognitive status in MS patients. 1 2 3

The alignment between the cognitive domains evaluated by the tests and the types of cognitive impairments commonly observed in MS makes it a valuable tool for disease monitoring and measuring treatment efficacy. Additionally, broader assessment tools are also extensively utilized in clinical practice, such as the MS Functional Composite (MSFC) battery, which evaluates cognitive, motor, and functional domains—including the Symbol Digit Modalities Test (SDMT) and the Paced Auditory Serial Addition Test (PASAT) for cognitive assessment. The SDMT primarily measures processing speed, one of the earliest and most commonly affected cognitive domains in MS. It also involves attention and working memory as secondary processes. 5 6 7

The Rey Complex Figure Test (RCFT) is a widely used neuropsychological tool for assessing cognitive domains such as visuospatial abilities, visual memory, and directed attention. 1 2 8 Previous studies have examined its performance in MS patients and provided normative data for the Turkish population, supporting its cross-cultural applicability in cognitive assessments. 8 9 10

The aim of this study was to compare cognitive performance assessed by RCFT and SDMT. Furthermore, understanding the relationships between these two tests, and of both with disability in RRMS patients, may provide critical insights into their contributions to clinical monitoring and treatment processes for cognitive impairments.

## METHODS

A total of 177 individuals, including 95 patients (73 female and 22 male) diagnosed with RRMS according to the 2017 McDonald criteria, aged 18 to 65 years, as well as 82 age and sex matched healthy volunteers (56 female and 26 male), were prospectively enrolled into the study. All patients were followed with a diagnosis of RRMS in the Neuroimmunology Clinic of the Kanuni Sultan Suleyman Training and Research Hospital, Istanbul, Turkey, between January 2025 and April 2025.

Those currently in an acute relapse or experienced a relapse within the last month were excluded. Our study was conducted in accordance with the Declaration of Helsinki's guidelines, and it was approved by the local ethics committee. All study procedures were approved by the Ethical Committee of the Kanuni Sultan Suleyman Training and Research Hospital, Istanbul, Turkey, under the protocol number KAEK/2024/11.261.

Demographic data (age, gender, marital status, education level) and disease-related characteristics of the patient group (disease duration, medications used for MS treatment, disease disability) were recorded. Disease disability was assessed using the Expanded Disability Status Scale (EDSS). In the present study, patients were assessed using the MSFC battery, which includes the SDMT, the Timed 25-Foot Walk (25FWT), and the 9-Hole Peg test (9HPT). The SDMT is a brief, widely used cognitive screening tool that evaluates processing speed, attention, and working memory. It was also evaluated independently as a measure of cognitive performance and compared with healthy controls.

The RCFT is a well-established tool for assessing visuospatial constructional ability, visual memory, and planning skills. The test consists of four stages:

- Copy (RCFT 0): Participants are asked to reproduce a complex figure presented on a card placed in front of them.
- Immediate Recall (RCFT 1): Immediately after the copy phase, participants are asked to draw the figure from memory, measuring short-term visual memory.
- Delayed Recall (RCFT 2): After a 20 to 30-minute interval, participants are once again asked to draw the figure from memory, assessing delayed visual memory and retention. Each drawing is scored on a 36-point scale based on the accuracy and placement of 18 figure components, following standardized scoring criteria.
- Recognition (RCFT 3): After delayed recall stage, participants are presented with an array of 24 figures containing components from the original complex figure, some of which are altered, mirrored, or spatially distorted. They are instructed to identify the exact elements that were part of the original figure. Performance is scored according to the number of correct identifications. This phase primarily measures cue-dependent retrieval and visual discrimination processes.

The SDMT is a brief, widely used cognitive screening tool that evaluates processing speed, attention, and working memory. Participants were instructed to match symbols to numbers based on a reference key within 90 seconds. The total score reflects the number of correct responses. Both tests were administered in a quiet environment by a trained neuropsychologist.

### Statistical analyses

Continuous variables were expressed as mean ± standard deviation (SD) or median and minimum-maximum values, while categorical variables were reported as frequency and percentages.

The Shapiro-Wilk test was used to assess normal distribution. The categorical variables were analyzed using the Fisher's exact and Pearson Chi-squared tests. For continuous variables, the Mann-Whitney U test was applied for non-normally distributed data, while independent t-test was used for normal distributions. Spearman's correlation test was employed to analyze the relationship between disease duration, EDSS, and other study parameters in the patient group.

Data were analyzed using the SPSS Statistics (IBM Corp.) version 23.0, with *p* -values < 0.05 being considered statistically significant. Given the exploratory design of the study, no correction for multiple comparisons was applied. Correlation results were interpreted cautiously to minimize the risk of type I error.

## RESULTS

The study included 177 individuals, of whom 95 were patients with MS and 82 were healthy controls.

There were no meaningful differences between the MS and control groups regarding age (MS: 35.81 ± 10.86 years, control: 34.98 ± 9.54 years; *p* = 0.754), weight (MS: 68.47 ± 12.20 kg, control: 70.21 ± 12.48 kg; *p* = 0.352), or height (MS: 165.60 ± 7.84 cm, control: 167.21 ± 8.29 cm; *p* = 0.187). In the MS group, the proportion of women was higher (76.8%) than in the control (68.3%); however, this was not a statistically significant difference ( *p* = 0.237).

In terms of smoking, 48.8% of the individuals in the control group were current smokers, while 41.1% in the MS group. Those who quit were included in the nonsmoker group. This was a nonsignificantly different comparison ( *p* = 0.379).

There were 92 (96.8%) patients using one of the Disease-Modifying Therapies (DMTs). The most frequently used current DMTs were natalizumab (28.4%), ocrelizumab (23.2%), fingolimod (15.8%), and dimethyl fumarate (12.6%).

Regarding chronic diseases, 8 MS patients (8.4%) had at least one comorbid condition, while 22% of control participants had chronic diseases. This difference was statistically significant ( *p* < 0.001). The most common comorbidities were diabetes mellitus in 4.2% of patients and hypertension in 3.2%.

When we compared the levels of education between the MS and control groups, we did not find any significant statistical difference among the different categories ( *p* > 0.05 for all). We did observe some small numerical variations. In the MS group, the number of participants who had completed only primary school was slightly higher (18.9%) than in the control group (9.8%; *p* = 0.093). The distribution of groups was comparable at the levels of secondary and high school education, with no significant differences ( *p* = 0.266 and 0.408, respectively). Although 52.4% of the participants in the control group held a university degree, compared to 45.3% in the MS group, this was not a statistically significant difference ( *p* = 0.368), as shown in Table 1 .

**Table 1:** Demographic characteristics of MS patients and healthy controls

		MS patients n = 95	Control group n = 82	*p* -value
**Age**	Mean ± SD Median (min–max)	35.81 ± 10.86 33.00 (16–63)	34.98 ± 9.54 33.00 (16–61)	0.754 a
**Female gender**	%	76.8	68.3	0.237 b
**Weight (kg)**	Mean ± SD Median (min–max)	68.47 ± 12.20 68.00 (44–98)	70.21 ± 12.48 68.00 (40–110)	0.352 c
**Height (cm)**	Mean ± SD Median (min–max)	165.60 ± 7.84 166.00 (150–185)	167.21 ± 8.29 168.00 (150–189)	0.187 c
**Smoking**	%	41.1	48.8	0.379 d
**Chronic disease**	n (%)	8 (8.4)	18 (22)	0.011 d
**Education level**	%			
Primary school		18.9	9.8	0.093 d
Secondary school		9.5	4.9	0.266 b
Highschool		26.3	32.9	0.408 d
University		45.3	52.4	0.368 d

Abbreviations: MS, multiple sclerosis; SD, standard deviation.

Notes: ^a^ Mann-Whitney U test; ^b^ Fisher's exact test; ^c^ Independent t-test; ^d^ Chi-squared test.

The mean EDSS score was 1.74 ± 1.47, with a median of 1.00 (range: 0–6), indicating that the sample overall had a relatively mild level of disability. Patients with an EDSS score of ≤ 3 accounted for 84.2% of the study population.

On the motor function front, the 25FWT, which assesses gait speed, had a mean completion time of 6.06 ± 1.98 (median: 6.03; range: 4–13.5) seconds. Fine motor skills of the dominant hand, as measured by the 9HPT, showed a mean of 21.62 ± 4.35 (median: 20.53; range: 16–38) seconds.

Among MS participants, the mean SDMT score was 36.81 ± 14.24, with a median of 37, ranging from 5 to 70.

Visual memory was assessed using the RCFT. Patients registered a mean score of 30.94 ± 3.75 (median: 32.00; range: 16–36) in the copy phase (RCFT 0). For immediate recall (RCFT 1), the mean score was 15.62 ± 6.96 (median: 16.00; range: 1–30), and for delayed recall (RCFT 2), the mean score was 15.99 ± 6.63 (median: 15.00; range: 4–29). These numbers indicate that while short-term encoding may remain relatively intact, there appear to be noticeable impairments in the MS population when it comes to recalling information after even a brief delay ( Table 2 ).

**Table 2:** EDSS, clinical and neuropsychological test results in MS patients

Test		MS patients
**Disease duration (years)**	Mean ± SD Median (min–max)	8.11 ± 6.93 7 (0–35)
**EDSS**	Mean ± SD Median (min–max)	1.74 ± 1.47 1.00 (0–6)
**EDSS ≤ 3**	%	84.2
**Current DMT** 1 = Interferon 2 = Glatiramer acetate 3 = Teriflunomide 4 = Dimethyl fumarate 5 = Fingolimod 6 = Natalizumab 7 = Ocrelizumab 8 = Cladribine 9 = Alemtuzumab 10 = None	%	2.1 3.2 4.2 12.6 15.8 28.4 23.2 7.4 0 3.2
**25FWT (sec)**	Mean ± SD Median (min–max)	6.06 ± 1.98 6.03 (4.0–13.5)
**9HPT – Dominant Hand (sec)**	Mean ± SD Median (min–max)	21.62 ± 4.35 20.53 (16–38)
**SDMT**	Mean ± SD (95% CI) Median (min–max)	36.81 ± 14.24 (33.91–39.71) 37 (5–70)
**RCFT**		
0	Mean ± SD (95% CI) Median (min–max)	30.94 ± 3.75 (30.18–31.70) 32.00 (16–36)
1	Mean ± SD (95% CI) Median (min–max)	15.62 ± 6.96 (14.20–17.04) 16.00 (1–30)
2	Mean ± SD (95% CI) Median (min-–max)	15.99 ± 6.63 (14.64–17.34) 15.00 (4–29)
3	Mean ± SD (95% CI) Median (min–max)	6.17 ± 1.89 (5.78–6.56) 6.4 (1.4–10.3)

Abbreviations: 25FWT, 25-Foot Walk Test; 9HPT, 9-Hole Peg Test; CI, confidence interval; DMT, Disease-Modifying Therapy; EDSS, Expanded Disability Status Scale; MS, multiple sclerosis; RCFT, Rey Complex Figure Test; SDMT, Symbol Digit Modalities Test; SD, standard deviation.

Notes: RCFT 0 - Copy, 1 - Immediate Recall, 2 - Delayed Recall, 3 - Recognition.

The RCFT's results demonstrated significant differences in visual memory performance between MS patients and healthy controls across all stages of the test. At the copy phase (RCFT 0), MS patients had a mean score of 30.94 ± 3.75, with a median of 32.00 (range: 16.0–36.0). In contrast, the control group had a mean score of 32.18 ± 3.11, median of 32.25 (range: 26.0–36.0). This discrepancy achieved statistical significance ( *p* = 0.036), suggesting that MS patients have somewhat impaired visual memory even at the very first encoding stage.

For the immediate recall phase (RCFT 1), which assesses the short-term retention of visual memories, the mean score for the group with MS was significantly lower, at 15.62 ± 6.96 (median: 16.00, range: 1.0–30.5), than the mean for the control group, which was substantially higher, at 20.07 ± 8.03 (median: 19.50, range: 5.0–33.0). This significant difference ( *p* < 0.001) indicates a pronounced decline in short-term visual memory among MS patients.

In the delayed recall phase (RCFT 2), reflecting longer-term visual memory retention, MS patients scored a mean of 15.99 ± 6.63, with a median of 15.00 (range: 4.0–29.0). The control group had a mean of 19.87 ± 8.14 and a median of 20.50 (range: 2.5–33.0). The observed difference was again statistically significant ( *p* < 0.001). This reinforces the idea that MS patients experience progressive impairment in visual memory retrieval over extended intervals.

In the recognition phase (RCFT 3), the mean score of the MS patients was 6.17 ± 1.89 (median: 6.40; range: 1.4–10.3), whereas the control group had a mean score of 7.76 ± 1.95 (median: 7.75; range: 2.9–11.6) with the difference being statistically significant ( *p* < 0.001), as shown in Table 3 .

**Table 3:** Comparison of RCFT performance between MS patients and control groups

		MS patients	Control group	*p* -value a
**SDMT**	Mean ± SD Median (min–max)	36.81 ± 14.24 37 (5–70)	44.71 ± 12.52 45 (7–71)	**< 0.001**
**RCFT**				
0	Mean ± SD Median (min–max)	30.94 ± 3.75 32.00 (16.0–36.0)	32.18 ± 3.11 32.25 (26.0–36.0)	0.036
1	Mean ± SD Median (min–max)	15.62 ± 6.96 16.00 (1.0–30.5)	20.07 ± 8.03 19.50 (5.0–33.0)	**< 0.001**
2	Mean ± SD Median (min–max)	15.99 ± 6.63 15.00 (4.0–29.0)	19.87 ± 8.14 20.50 (2.5–33.0)	**< 0.001**
3	Mean ± SD Median (min–max)	6.17 ± 1.89 6.4 (1.4–10.3)	7.76 ± 1.95 7.75 (2.9–11.6)	**< 0.001**

Abbreviations: MS, multiple sclerosis; RCFT, Rey Complex Figure Test; SD, standard deviation.

Notes: ^a^ Mann-Whitney U test. RCFT 0 - Copy, 1 - Immediate Recall, 2 - Delayed Recall, 3 - Recognition.

A significant positive correlation was found between the 9HPT and EDSS (r = 0.373; *p* < 0.001). This means that disability on the EDSS is associated with slower peg test times. No significant correlations were observed between the 9HPT with other clinical or cognitive parameters.

Also, a substantial negative connection was discovered to exist between EDSS and SDMT scores (r = −0.330; *p* = 0.001). This indicates that disability is associated with slower information processing speed.

Conversely, there were no significant correlations found between the EDSS and either the 25FWT (r = 0.049; *p* = 0.635) or the scores from RCFT 0 (r = −0.165; *p* = 0.11), 1 (r = −0.086; *p* = 0.407), and 2 (r = −0.031; *p* = 0.762), as shown in Table 4 .

**Table 4:** Correlations between EDSS and cognitive/motor tests

Test	Spearman's rho	*p* -value
**25FWT**	0.049	0.635
**9HPT**	0.373	< 0.001*
**SDMT**	−0.330	0.001*
**RCFT**		
0	−0.165	0.11
1	−0.086	0.407
2	−0.031	0.762
3	–0.000	0.974

Abbreviations: 25-Foot Walk Test, 25-Foot Walk Test; 9HPT, 9-Hole Peg Test; EDSS, Expanded Disability Status Scale; RCFT, Rey Complex Figure Test; SDMT, Symbol Digit Modalities Test.

Notes: RCFT 0 - Copy, 1 - Immediate Recall, 2 - Delayed Recall, 3 - Recognition.

The relationship between the SDMT and the four components of the RCFT was examined in this study using Spearman correlation analysis. Results indicated that SDMT scores were significantly and positively correlated with the first three components of the RCFT. A moderate positive correlation was found between SDMT and RCFT 0 (rho = 0.408, *p* < 0.001), as well as a moderate and positive correlation with both RCFT 1 (rho = 0.433; *p* < 0.001) and 2 (rho = 0.445; *p* < 0.001), as shown in Table 5 .

**Table 5:** Correlations between SDMT and RCFT scores

RCFT	Spearman's rho	*p* -value
0	0.408	**< 0.001**
1	0.433	**< 0.001**
2	0.445	**< 0.001**
3	−0.040	0.67

Abbreviations: RCFT, Rey Complex Figure Test; SDMT, Symbol Digit Modalities Test.

Notes: RCFT 0 - Copy, 1 - Immediate Recall, 2 - Delayed Recall, 3 - Recognition.

The relationships between the 25FW and the 9HPT and the RCFT subtests were also evaluated using Spearman correlation analysis. No significant correlation was found with the 25FWT. In contrast, significant negative correlations were observed between 9HPT and RCFT subtests. A moderate negative correlation was found between 9HPT and RCFT 0 (rho = −0.350, *p* = 0.001), 1 (rho = −0.325; *p* = 0.001), and 2 (rho = −0.380; *p* < 0.001).

## DISCUSSION

Cognitive impairments in MS commonly affect attention and executive function, even in the early stages of the disease. Additionally, visuospatial and visual memory deficits linked to structural and connectivity changes in the hippocampus, precuneus, and parietal regions are increasingly recognized as important contributors to functional decline. These regions play a critical role in both spatial memory and imagery processes that require complex visual information integration. 11

This study confirms that RRMS significantly affects two aspects of cognition: visual memory and visual-structural skills, as assessed by the RCFT; and processing speed, as measured by the SDMT. Consistent with this framework, our results demonstrated that these patients showed impairments in visuospatial memory (RCFT) and processing speed (SDMT) compared with healthy controls.

Dimitrov et al. demonstrated in their study involving 70 patients with MS and 32 healthy controls that the RCFT was able to detect significant cognitive differences between the groups. 12 The MS group performed significantly worse than the control group in the shape copying task (mean score: 33.3 ± 6.3 vs. 35.4 ± 1.3; *p* < 0.05) and the memory recall task (mean score: 19.7 ± 7.2 vs. 24.5 ± 6.7; *p* < 0.01) compared to the control group. Additionally, the shapes copied by MS patients were significantly more distorted than those identified in previous cognitive dysfunction measurements ( *p* < 0.001).

Another notable finding of our study is the moderate correlation we found between SDMT and RCFT 0, 1, and 2 scores. These tools appear to measure some common cognitive domains such as attention, processing speed, and working memory. 2 9 13 Undoubtedly, the SDMT is known as an extremely sensitive tool that reveals the first signs of cognitive decline in MS patients. 14 It primarily assesses a person's speed of processing simple visual stimuli, but does not even begin to address the complex, multidimensional, visual-sensory problems, as posed by the RCFT's tasks. If the SDMT indicates that a person is beginning to decline, the RCFT can provide a more detailed understanding of specific cognitive processes.

Additionally, research conducted by Wang et al. on healthy older adults has shown that performance on the RCFT 1 test is positively related to learning new skills and motor sequence learning, reinforcing the idea that this task is useful for assessing more executive-like processes related to learning. 15 Although this finding is not specific to the MS population, it supports the applicability of this test as a measure of cognitive performance.

Interestingly, RCFT 3 (recognition phase) demonstrated a significant difference between MS patients and healthy controls, yet it did not correlate with SDMT scores. This suggests that recognition-based visual memory, which relies on cue-assisted retrieval and visual discrimination, may depend on partially distinct cognitive mechanisms from those captured by processing speed tasks. The presence of visual cues in recognition tasks likely reduces attentional and executive demands, which may explain why patients' performance can be preserved relative to recall in those with slower processing speed.

Our study had a relatively low average EDSS score of 1.74 ± 1.47. This indicates that the average patient has very little-to-mild, if any, physical disability. The predominant impairment observed in these patients was cognitive, and particularly severe impairment is present in visual memory and delayed recall. This inconsistency supports the widespread perception that cognitive impairment in MS can develop independently of physical disability. 10 Indeed, early cognitive decline, particularly in processing speed and executive function, has long been recognized as a symptom even in the earliest stages of MS. 16

The lack of physical and cognitive correlation in degree of impairment is similar to what Inglese et al. mention in their study of MS using diffusion tensor magnetic resonance imaging (MRI). 17 They have shown that this cohort has widespread axonal damage in normal-appearing white matter where motor functions are preserved. Essentially, they argue that even if motor function pathways are not significantly damaged, increasing microstructural changes may affect cognitive functions. Their findings suggest that a large number of our participants had very much preserved motor function, albeit with significant and profound cognitive impairments that don't seem to show up on the EDSS scale. 17

Pitteri et al. 18 conducted an 8-year prospective study that showed that initial cognitive impairment in RRMS patients was predictive of future disability progression and cortical thinning. Interestingly, there was a group of patients with relatively low EDSS scores at baseline (e.g., 2.0–3.6), who experienced significant clinical and structural deterioration over time, especially if they had cognitive impairments. 18 The findings from these studies reinforce the idea that early cognitive dysfunction in MS is not only common but also prognostically significant.

Furthermore, the lack of a strong correlation between RCFT scores and EDSS in our data is consistent with a previous study by Jakimovski et al., 19 which found that EDSS primarily reflects motor impairment but may not reflect widespread microstructural damage that broadly affects cognitive function. Similarly, Matías-Guiu et al. noted that delayed recall and executive function impairments are associated with atrophy in specific brain regions (e.g., thalamus, caudate, and precuneus) rather than overall brain lesion burden. 20 In line with this framework, our results suggest that the visual memory impairments exhibited by MS patients (observed in all stages of the RCFT) may be more closely related to damage affecting the subcortex and posterior cortex than to brain damage with visible effects, such as motor impairments that would result in a low EDSS score.

This study has some limitations. Interrater reliability was not tested, and the single-center design may have introduced sampling bias. Therefore, caution is needed when generalizing these results, which are specific to RRMS patients with mild disability to progressive forms of MS, or more diverse patient populations. As several correlations were explored, corrections for multiple comparisons were not applied. Thus, the possibility of Type I error should be considered when interpreting the results.

Furthermore, since the design was cross-sectional, it does not allow for conclusions about the progression of cognitive impairments over time. Although cognitive impairment in MS is often linked to structural brain changes, such as cortical atrophy or lesion load, no MRI-based structural or functional correlates were analyzed in the present study.

While the total number of participants was moderate, the sample size was insufficient for subgroup analyses as EDSS and education level. Information on visual acuity, ocular motor function, and history of optic neuritis was not available for all participants, which may have influenced performance on visually demanding tests such as RCFT and SDMT. Mood, fatigue, and medication effects were not systematically evaluated and may have influenced cognitive performance. Although educational level was recorded categorically, years of formal education were not available, which limits the ability to control for its influence on cognitive performance.

The Z-scores could not be calculated because standardized age- and education- adjusted normative data were not available for all RCFT subtests and SDMT reference sets.

This study highlights the distinct yet complementary roles of the RCFT and the SDMT in evaluating cognitive dysfunction in patients with RRMS. Our findings demonstrate that patients with MS show significant impairments not only in processing speed, as measured by the SDMT, but also in immediate and delayed visual memory, as assessed by the RCFT. These deficits were evident even in patients with low EDSS scores, highlighting the dissociation between physical disability and cognitive decline in MS.

Furthermore, RCFT 0, 1, and 2 showed moderate correlations with SDMT scores, while 3 (recognition) revealed a significant difference between MS patients and healthy controls but did not correlate with the test. This suggests that recognition-based visual memory engages partially distinct cognitive mechanisms that are less dependent on processing speed. Incorporating both recall- and recognition- based measures into assessments may provide a more comprehensive understanding of cognitive strengths and weaknesses in MS.

Taken together, these findings suggest that RCFT may provide complementary information to SDMT in the cognitive assessment of MS patients, particularly in domains involving memory and organization, without implying routine clinical use or superiority over other widely used tests.

## References

[1] Eijlers A JC van Geest Q Dekker I Predicting cognitive decline in multiple sclerosis: a 5-year follow-up study Brain 2018 141 09 2605 2618 10.1093/brain/awy202 30169585

[2] Kania K Pawlak M A Forycka M Predicting clinical progression and cognitive decline in patients with relapsing-remitting multiple sclerosis: a 6-year follow-up study Neurol Neurochir Pol 2024 58 02 176 184 10.5603/pjnns.97714 38324117

[3] Marasescu R Garcia M C Benito Y A Impairment of visuospatial/visuoconstructional skills in multiple sclerosis patients: the correlation with regional lesion load and subcortical atrophy Neurologia 2016 31 03 169 175 10.1016/j.nrl.2015.06.003 26342250

[4] Ozben S Kucuksayan E Koseoglu M Erel O Neselioglu S Ozben T Plasma thiol/disulphide homeostasis changes in patients with relapsing-remitting multiple sclerosis Int J Clin Pract 2021 75 07 e14241 10.1111/ijcp.14241 33891773

[5] Benedict R HB Smerbeck A Parikh R Rodgers J Cadavid D Erlanger D Reliability and equivalence of alternate forms for the Symbol Digit Modalities Test: implications for multiple sclerosis clinical trials Mult Scler 2012 18 09 1320 1325 10.1177/1352458511435717 22277740

[6] MacAdam E S Berard J A Walker L AS Cognition and Cognitive Fatigability: Association with Employment Status in Multiple Sclerosis Can J Neurol Sci 2023 50 06 870 875 10.1017/cjn.2022.309 36280897

[7] Yaldizli Ö Penner I K Frontzek K The relationship between total and regional corpus callosum atrophy, cognitive impairment and fatigue in multiple sclerosis patients Mult Scler 2014 20 03 356 364 10.1177/1352458513496880 23959709

[8] Dinn A A Dinn W M Rey Complex Figure Test Profile of Turkish Adults Archives of Neuropsychiatry 2012 49 145 151 10.4274/npa.y6306

[9] Varan EÖ Tanör ÖÖ Gürvit H Rey Complex Figure Test and Recognition Trial (RCFT): Norm Determination Study on Turkish Adult Sample Turk J Neurol 2007 13 06 387 394

[10] German Competence Network Multiple Sclerosis (KKNMS) Johnen A Bürkner P C Landmeyer N C Can we predict cognitive decline after initial diagnosis of multiple sclerosis? Results from the German National early MS cohort (KKNMS) J Neurol 2019 266 02 386 397 10.1007/s00415-018-9142-y 30515631 PMC6373354

[11] Chiaravalloti N D Genova H M DeLuca J Cognitive rehabilitation in multiple sclerosis: the role of plasticity Front Neurol 2015 6 67 10.3389/fneur.2015.00067 25883585 PMC4383043

[12] Dimitrov I Kirkova V Kaprelyan A Application of the Rey-Osterrieth complex figure test for assessment of cognitive impairment in multiple sclerosis Scripta Scientica Medica 2015 47 03 59 64 10.14748/ssm.v47i3.1303

[13] Park J Y Seo E H Yoon H J Won S Lee K H Automating Rey Complex Figure Test scoring using a deep learning-based approach: a potential large-scale screening tool for cognitive decline Alzheimers Res Ther 2023 15 01 145 10.1186/s13195-023-01283-w 37649070 PMC10466875

[14] Benedict R HB Amato M P DeLuca J Geurts J JG Cognitive impairment in multiple sclerosis: clinical management, MRI, and therapeutic avenues Lancet Neurol 2020 19 10 860 871 10.1016/S1474-4422(20)30277-5 32949546 PMC10011205

[15] Wang P VanGilder J L Schweighofer N Schaefer S Y Rey-Osterrieth complex figure recall scores and motor skill learning in older adults: A non-linear mixed effect model-based analysis Hum Mov Sci 2022 86 103004 10.1016/j.humov.2022.103004 36191575 PMC11285843

[16] Anhoque C F Biccas L Neto Domingues S CA Teixeira A L Domingues R B Cognitive impairment in patients with clinically isolated syndrome Dement Neuropsychol 2012 6 04 266 269 10.1590/S1980-57642012DN06040011 29213807 PMC5619339

[17] Inglese M Adhya S Johnson G Perfusion magnetic resonance imaging correlates of neuropsychological impairment in multiple sclerosis J Cereb Blood Flow Metab 2008 28 01 164 171 10.1038/sj.jcbfm.9600504 17473851 PMC2596621

[18] Pitteri M Romualdi C Magliozzi R Monaco S Calabrese M Cognitive impairment predicts disability progression and cortical thinning in MS: An 8-year study Mult Scler 2017 23 06 848 854 10.1177/1352458516665496 27527906

[19] Jakimovski D Zivadinov R Ramanthan M Serum neurofilament light chain level associations with clinical and cognitive performance in multiple sclerosis: A longitudinal retrospective 5-year study Mult Scler 2020 26 13 1670 1681 10.1177/1352458519881428 31610732

[20] Matías-Guiu J A Cortés-Martínez A Montero P Identification of Cortical and Subcortical Correlates of Cognitive Performance in Multiple Sclerosis Using Voxel-Based Morphometry Front Neurol 2018 9 920 10.3389/fneur.2018.00920 30420834 PMC6216547

